# A Health Professional–Led Synchronous Discussion on Facebook: Descriptive Analysis of Users and Activities

**DOI:** 10.2196/formative.7257

**Published:** 2017-11-22

**Authors:** Rebecca Grainger, Bonnie White, Catherine Morton, Karen Day

**Affiliations:** ^1^ Rehabilitation Research and Teaching Unit Department of Medicine University of Otago Wellington Wellington South New Zealand; ^2^ Hutt Hospital Hutt Valley District Health Board Lower Hutt New Zealand; ^3^ Arthritis Foundation of New Zealand Wellington New Zealand; ^4^ School of Population Health The University of Auckland Auckland New Zealand

**Keywords:** social media, arthritis, patient education, patient engagement

## Abstract

**Background:**

Arthritis is a major cause of pain and disability. Arthritis New Zealand (Arthritis NZ) is a nongovernmental organization that provides advocacy, information, and advice and support services for people with arthritis in New Zealand. Since many people seek health information on the Web, Arthritis NZ has a webpage and a Facebook page. In addition to static content, Arthritis NZ provides synchronous discussions with an arthritis educator each week via Facebook.

**Objective:**

The aim of this study was to describe participation and structure of synchronous discussion with a health educator on a social media platform and the type of information and support provided to people with arthritis during discussions on this social media platform.

**Methods:**

Interpretive multimethods were used. Facebook Analytics were used to describe the users of the Arthritis NZ Facebook page and to provide descriptive summary statistics. Graphic analysis was used to summarize activity during a convenience sample of 10 arthritis educator–led synchronous discussions. Principles of thematic analysis were used to interpret transcripts of all comments from these 10 weekly arthritis educator–led discussions.

**Results:**

Users of the Arthritis NZ Facebook page were predominantly female (1437/1778, 80.82%), aged 18 to 54 years. Three major activities occurred during arthritis educator–led synchronous discussions: (1) seeking or giving support; (2) information enquiry; and (3) information sharing across a broad range of topic areas, largely related to symptoms and maintaining physical functioning. There was limited peer-to-peer interaction, with most threads consisting of two-comment exchanges between the users and arthritis educators.

**Conclusions:**

Arthritis educator–led discussions provided a forum for informational and emotional support for users. The facilitated discussion forum for people with arthritis on Facebook could be enhanced by encouraging increased user participation and increasing peer-to-peer interactions and further training of arthritis educators in facilitation of Web-based discussion. Future research should focus on addressing barriers to user participation and assessing the impact of arthritis educator facilitation training, with the latter leveraging the Action Research paradigm.

## Introduction

People need more than medical diagnosis and intervention to be able to live well while affected by arthritis. Arthritis is the single greatest cause of disability in many parts of the world, affecting 13% to 28% of people, with the burden expected to increase with the aging of developed populations [1]. In the United States, the total financial cost of musculoskeletal diseases, most of which is arthritis, was estimated at US $926 billion in 2011, which was 5.7% of the gross domestic product [2]. Although arthritis is rarely fatal, it has no cure and can affect function and quality of life [3,4], which is reflected in the high indirect financial costs that include loss of employment, informal care, aids, and additional costs when traveling. Difficulties with daily tasks [5] may cause psychological distress for people living with arthritis and their families and carers [6].

In New Zealand, citizens and permanent residents are eligible for free medical treatment in public hospitals, whereas other health services and medicines are subsidized. Primary care physicians (general practitioners [GPs]) are the gatekeepers to medical specialists and surgeons, and people must be referred by their GP to see a specialist. Specialist care usually focuses on medical or surgical management, with less attention on the psychological and functional impacts of arthritis. Appointments are short (usually 15-min long) and clinicians often use medical jargon, and thus people affected by arthritis may end up feeling marginalized, which causes further psychological distress [7-9]. Nongovernmental organizations such as Arthritis New Zealand (Arthritis NZ) provide information, advice, and support services to people with arthritis to supplement medical care [10].

Arthritis NZ’s mission is “to improve the lives of people living with arthritis.” Its key program areas include advocacy, awareness, information and advice services, research, and support services [11]. The information and advice and support services have traditionally been provided to individuals, groups, and communities by arthritis educators, who are generally nurses or allied health professionals employed by Arthritis NZ, in person and by telephone.

The Internet age has impacted how people seek information and interact with one another about and in response to information they find. Nearly 80% of people in high-income countries access the Internet for more than 1 hour each day [12-14]. Social networking sites are a key activity [12,15]. Since so many people use the Internet to seek health-related information [16,17], many health organizations now have a significant presence on the Web. Support groups meeting the needs of people with long-term health issues, such as diabetes and arthritis, have become popular on Facebook [18]. Social media, in the form of online discussion forums, offer people the opportunities to retrieve, share, and exchange information and experiences, find meaning, and help others [19]. It is flexible in terms of synchronous and asynchronous communication, regardless of geography, time zone, or health system in which participants (active or lurking) usually operate. Informational support, as defined by Cohen and Wills [20], is readily available on the Web but is largely unpredictable and the benefits, risks, and affordances unverified [18,19].

In 2013, Arthritis NZ started a Facebook page for staff and consumers to communicate by leaving posts about topics of interest. A unique feature is the weekly live, synchronous, 2-hour long discussion session led and moderated by an arthritis educator. People join the conversation by clicking on the like button on the Arthritis NZ Facebook page and receive passive updates in their own Facebook newsfeed. They can read postings on the Arthritis NZ page and respond if they wish. The Arthritis NZ Facebook page thereby provides synchronous and asynchronous informational support in the form of an online forum for people affected by arthritis to connect with an arthritis educator and others like them. Similar online communities have been shown to support reciprocal information sharing and facilitate patients moving from simple information gathering to behavioral change [18,19,21]. Although some research has been conducted on the use of asynchronous communication on Facebook for informational support, no research has been done on how synchronous, live, moderated discussions work for supporting people living with arthritis.

The aim of this research project was to conduct an analysis of participants, use, and content of the Arthritis NZ Facebook discussion service to describe the demographics of users of the Arthritis Facebook page and the structure of, and participation in, synchronous discussions with a health educator on the page and the type of information and support people are seeking when participating in these conversations on this social media platform.

## Methods

### Study Setting and Demographics of the Arthritis NZ Facebook Page Users

Since 2013, a banner on the Arthritis NZ Facebook page stated that every Monday night, between 7:30 PM and 9:30 PM, an arthritis educator would be available to post answers to questions on the Facebook page. Arthritis educators were aware that their role was to provide information and advice, moderate any comments to ensure that the content remained constructive, correct errors in users’ posts, and redirect discussions back on topic if the need arose. Arthritis educators had not received any training in communication in social media or online fora. All discussion sessions were run by arthritis educators except for the final session, which was run by the leader of the Arthritis NZ advocacy program.

Data were collected from the Arthritis NZ Facebook page by a member of the research team who was also an employee of Arthritis NZ (CM). A quantitative analysis of the Arthritis NZ Facebook page users was conducted using Facebook Analytics. The data were extracted using the Page Insights function of Facebook on December 7, 2015.

### Discussion Participation

All content related to the 10 Monday nights, arthritis educator–led synchronous discussions, between October 12 and December 21, 2015, was copied and pasted into “transcripts” in Microsoft or MS Word documents by CM. Updates and other posts by Arthritis NZ that occurred outside these sessions were excluded. Each transcript replicated the structure of the discussion thread, including the content, coded names of participants, and frequency and placement of “likes.” The data included all posts by page users or arthritis educator on duty during the synchronous discussion session (short updates, questions, and comments), comments (in response to a post by anyone), and likes. Page activity data were summarized using counts and descriptive statistics, including the number of participants, the number of posts (comments), conversations (a post on a new topic with no reference to previous posts with the subsequent posts directly in reply to the new topic), replies (a comment replying directly to a previous post or comment), the frequency of contributions by participants, and who conversed with whom about what. The open-source Gephi computer program [22] was used to describe the network of active users (people who had commented on the Arthritis NZ Facebook page). A simple diagrammatic analysis of 2 discussions was also constructed.

### Discussion Thread Content

The data were analyzed by reading the transcripts repeatedly, identifying codes that clustered into themes, and reviewing and naming the themes [23-25]. Each transcript of qualitative data was printed, cut, and coded, and then manually grouped by theme, for example, information seeking. Transcripts were kept as MS Word documents and imported into an MS Excel file for detailed coding and analysis. Each transcript was analyzed as a sequence of comments (ie, as it appeared in the Arthritis NZ Facebook page). Memos were written about the codes to enhance the analysis. KD coded the first 2 transcripts to set up the basic parameters of the analysis. BW then coded all the transcripts, adding details for the codes. Once all the transcripts were coded, RG independently coded 2 weeks’ randomly selected transcripts. Where uncertainty or differences in coding occurred, a discussion was held to achieve convergence. The coding aligned for all 3 researchers by the end of the analysis.

### Ethical Considerations

This study was designed in collaboration with the Arthritis NZ management team, principally the Senior Advisor of Clinical Services and Research (CM). Ethical approval was obtained through the University of Otago Human Ethics Committee (OUHEC (Health) D15/316). The Chief Executive of Arthritis NZ provided written informed consent on behalf of Arthritis NZ, and all arthritis educators also provided written consent for participation. Arthritis educators were able to opt out of participation with no consequence from their employer. A pinned post was displayed at the top of the Facebook page throughout the study period, informing viewers that page activities were being collected and anonymized for research. A link to the full participant information sheet and opt-out mechanisms was provided. An Arthritis NZ employee (CM) collected the data, anonymized it for analysis purposes and to protect the identity of the Facebook page users, and sent it to the researchers for analysis. The discussion threads have been deleted in Facebook to avoid participants from being identified. The pinned post and participant information sheet are available in Multimedia Appendix 1.

## Results

### Demographics of the Arthritis NZ Facebook Page Users

On October 12, 2015 (start of study period), the Arthritis NZ Facebook page indicated that 1778 people had clicked the like icon and were therefore users of the page. As on December 7, 2015, the majority were females (80.82%) who were in the age group of 25 to 54 years (Table 1). In a 4-week period within the study period (November 7 to December 7, 2015), only 22.05% (392/1778) of users clicked on any aspect of the Facebook page, and 8.38% (149/1778) actively participated on the page (ie, posted to the timeline, commented or shared a page post, or responded to a posting).

### Discussion Participation

Arthritis educator–led discussion threads were relatively small, with a median of 7 users (excluding arthritis educator) posting (range 2-27) a median of 5.5 conversations (range 1-24) and a median of 25.5 posts (range 10-77) (Table 2). Arthritis educator–led sessions for weeks 1 to 9 of the study were of similar size, with a median of 22 comments and 7 users. The final arthritis educator session was facilitated by the leader of the Arthritis NZ advocacy program rather than an arthritis educator and was larger with 27 users contributing 77 comments.

**Table 1:** Demographics of likers of Arthritis New Zealand Facebook page.

Age in years	Users n (%)	Female n (%)	Male n (%)
13-17	19 (1.06)	15 (0.85)	4 (0.23)
18-24	160 (8.99)	124 (6.97)	36 (2.02)
25-34	391 (21.99)	320 (17.99)	71 (3.99)
35-44	427 (24.01)	356 (20.02)	71 (3.99)
45-54	373 (20.97)	302 (16.98)	71 (3.99)
55-64	249 (14.00)	213 (11.98)	36 (2.02)
65+	125 (7.03)	107 (6.01)	18 (1.01)
Total	1778 (100)	1437 (80.82)^a^	307 (17.27)^a^

^a^34 users did not state gender.

**Table 2:** Quantitative descriptors of all arthritis educator discussion sessions.

Quantitative descriptors	Number of active participants (including arthritis educators)	Number of conversations	Number of comments
Mean	8.6	7.4	29.5
Median	7	5.5	25.5
Range	2-27	1-24	10-77
Total	55	74	295

This may be because the Arthritis NZ advocacy leader asked for comments on areas that users wished for advocacy with government agencies or the health system. Furthermore, 14 users replied to other users during this final session, whereas 11 users had replied to others during weeks 1 to 9 combined.

Arthritis educator comments accounted for 44.1% (130/295) of all comments over the 10-week study period. Excluding arthritis educators, most users commented infrequently. In total, there were 55 individuals who posted in discussion threads: 60% (33/55) posted in only 1 conversation, 25% (14/55) posted in 2 conversations, 9% (5/55) posted in 3 conversations, 4% (2/55) posted in 4 conversations, and 2% (1/55) posted in 5 conversations. The 10 users who commented most frequently contributed 48.1% (142/295) of all comments. Most comments were new conversations, with only 28.1% (83/295) of comments being in reply to another user’s comment.

**Figure 1:**
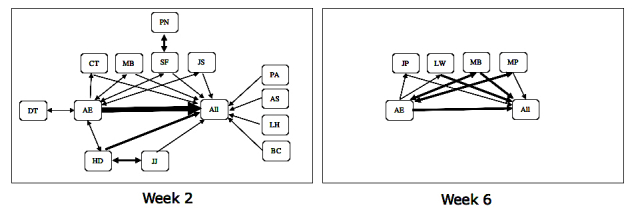
Manual network analysis of two discussion threads.

The network diagram generated by Gephi did not reveal a meaningful analysis other than that one person (possibly arthritis educator) contributed to the bulk of the postings and most participants responded to those posts rather than to one another, with a few people responding to one another. Two weeks’ transcripts were selected and manually analyzed, as depicted in Figure 1, because they represent different forms of conversation (one with many participants and one with few). The darker the lines between participants, the more comments attributed to their interaction. An arthritis educator always opened the discussion with an invitation and closed the session with a closing comment. The arthritis educator responded to topic initiators, and, in many instances, a combination of interactions between the initiator and arthritis educator ensued with some participants contributing sometimes. This pattern where an arthritis educator dominated the conversation was apparent in the transcripts of all arthritis educator–led discussion sessions.

More people participated in week 2 (n=13) than week 6 (n=4), when the interaction was predictably simpler. In both weeks, the arthritis educator directed most postings to “all,” and people initiated topics to “all.” There were 3 conversations that did not appear to involve “all” in week 2 and none in week 6. Furthermore, it appears that when a participant posts a comment to “all,” responses are directed to specific people. For example, in week 6, MB posted a question about shin splints and a conversation consisting of 4 comments ensued between MB and the arthritis educator.

### Discussion Thread Content

Three themes were identified in the content of the comments. The major theme was seeking and giving support, through sharing of experiences (from other users), or information (usually from arthritis educators). Two smaller themes of information inquiry and information sharing were also identified.

#### Theme 1: Seeking and Giving Support

Comments seeking support included expressions of negative impacts of arthritis regarding symptoms, emotional well-being, daily function, and participation. Users’ comments indicated how the diagnosis of arthritis often invoked fear, uncertainty, and isolation, as described below:

It's terrifying for me I got told I have psoriatic arthritis and handed some steroid meds [sic] and a pamphlet on methotrexate which they told me to go on it’s a scary, scary sounding medication.

They felt they were on their own and perceived a lack of emotional support from the health system. They expressed frustration and dissatisfaction with health care services.

Suboptimal symptom control, particularly pain, impacted on emotional well-being, with one participant commenting:

...sometimes it’s hard to stay positive when even getting pain relief is an issue.

Comments on impaired function and social participation referenced the social construct of disability [26]. For example, use of crutches or wheelchairs and limited parking space restricted access to public spaces. Some participants described how workplaces may not be supportive of people with arthritis, which could have an impact on work participation:

Many workplaces/managers think it's just too hard to employ someone with a chronic disease and say it's a performance issue when they take sick leave.

Although public hospital specialist services are free at the point of care in New Zealand, a combination of financial difficulties and appointment waiting times was a common barrier to health care service access. One participant asked for help with shin splint treatment, saying that she “can’t afford to buy expensive runners…” Another participant said:

My doc [GP] referred me to the public hospital but they don't want to know. I do have [insurance] but as I am a single working mum finding the extra 20% is just unobtainable...

Several participants commented about their perceptions about their doctors’ knowledge and lack of emotional support. One of the participants said:

...my doc doesn’t seem to care.

Another participant observed that her doctor’s focus was “usually diagnosis, drugs, and off you go” when she felt a need for her doctor to provide support group information.

Support was given by (1) the Arthritis NZ arthritis educators and (2) sought from participants by others, in arthritis educator–led discussions on Facebook. Arthritis educators offered emotional support with positive feedback and endorsed constructive lifestyle changes. Informational support included advice about nonpharmacological management options, strategies for coping and promoting emotional well-being, and suggestions about the most appropriate health care professionals for specific concerns. This quote below from an arthritis educator is representative of the type and depth of informational advice provided:

Fatigue is such a common symptom, even when you are not in a “flair”[sic] period of RA. It is certainly worth investigating to see if there is any underlying reason like anaemia. However pacing, regular moderate exercise, and dedicated time for relaxation may help. Low energy is one of the possible side effects of methotrexate. There are other hints about fatigue that might help, one of the educators could have a chat if you want to give us a call.

Participants acknowledged informational support from Arthritis NZ but expressed a need for more emotional support. They also expressed a preference for peer-to-peer emotional support, which could occur on the Web. In contrast to their expressed desire, direct participant-to-participant expressions of emotional support were infrequent, as can be seen in the interaction pattern in Figure 1. A participant indicated that Arthritis NZ provides good informational support and that she looks to other online groups for emotional support:

I’ve found online groups way better in terms of support so google [sic] them and make contact.

#### Theme 2: Information Inquiry

Participants requested information for four key reasons. The first reason was to obtain information to contextualize their symptoms and/or comorbidities in relation to arthritis or its treatments, and they were looking for explanations of what they were experiencing. For example, “Is arthritis worse in cold weather?” One person wanted to know about the relationship between inflammation and “a burning in the knee” and another had no pain issues but was on treatment for “wicked acid attacks” and was looking for ways to augment the medication already being taken.

The second reason for information inquiry was to find solutions to mitigate functional impairments (eg, access in and out of cars, walking shoes, and packaging). One participant wanted to do some walking and was looking for a recommendation on “good men’s shoes that are supportive for arthritis that is causing inflammation in the Achilles.”

The third reason for information inquiry was to understand the usefulness or implementation of lifestyle changes such as diet, improving sleep quality, and benefits or harm from complementary or alternative therapies.

Finally, they sought knowledge to optimize their experience and understanding of medical care, including recommendations for knowledgeable or sympathetic doctors. They asked for information about medication use, including side effects of arthritis-specific medications (eg, methotrexate and biologic disease modifying antirheumatic drugs) and the utility of deferring medication recommended by their rheumatologist until their personally assessed need exceeds their perceptions of harm, as expressed below:

I have not been taking any meds and do as much as I can but recently one knee has been giving me lots of grief with aching, locking and feeling very swollen...I guess my question is what sorts of meds should I look into to help me? I haven’t wanted to start on meds until really needed.

#### Theme 3: Information Sharing

Shared information included triggers for symptoms and experiences of symptom management strategies. Arthritis educator offered most information, often detailing scientific rationales for treatments and providing external links to Web-based information. Almost half of the comments by arthritis educators offering information (18 out of 45 comments) recommended consultation with a doctor, and 8 out of 45 comments recommended consulting another health professional (including physiotherapist, dietician, pharmacist, nurse, and podiatrist). These comments are recognizing the limitations of their own professional boundaries and the online environment. When participants offered solutions to others, more conversation was stimulated. Solutions offered included nonpharmacological treatment (eg, exercise, weight loss), advice to consider surgery, and alternative approaches to completing activities of daily living. One participant described her exploration of yoga, saying:

Did my first yoga class last week was a bit worried whether my RA joints and body would cope but it was brilliant. The graceful stretching and meditation was amazing but it certainly made me realise how tight my body gets from holding pain all the time. Really think yoga might be my thing for helping me relax and destressing.

## Discussion

### Principal Findings and Comparison With Prior Work

The Arthritis NZ Facebook likers were predominantly younger women. This is consistent with other data showing that women seek health information on the Web more often than men, are more likely to use social media and blogging for health reasons, and have a lower dropout rate in Web-based, self-help interventions compared with men [13,27-29].

Arthritis educator–led synchronous discussions were small, with a median of 8 participants. Only 5% of the likers of the Arthritis NZ Facebook page posted comments during the 10 discussions, with 10 users contributing half of all comments. A similar public question-and-answer session on Facebook for medication use questions also reports low number of active comments with a mean of 5 questions per hour; however, extensive shares and likes resulted in a mean reach of 3776 per week, suggesting the information was useful to a much larger number of people [30]. These observations are consistent with other Web-based behavior; less than 20% of people who read other people’s health experiences actually posted health-related comments themselves [31]. Those who lurked probably benefited from reading information in online support groups, but sharing information in online support groups is more effective in enhancing mental and social well-being [32,33].

The key activity identified in arthritis educator–led discussions was seeking or giving support, the majority of which was informational support although instances of emotional support occurred. Content analysis of Facebook groups for diabetes [18,34] and dialysis [35] has shown information sharing and emotional support as the predominant activities on these pages. These Facebook groups were not moderated or supported by a health organization, and therefore, also contained promotion of non–FDA (Food and Drug Administration)-approved treatments [18], and health advice was provided by peers rather than health professionals [18,34,35]. The analysis of the synchronous arthritis educator–led discussions confirmed that a synchronous discussion on a social media platform can be used to provide health information relevant to an individual’s requirements, as previously observed [27]. Furthermore, arthritis educators, who also recognized limitations of providing information on the Web without context, recommended individuals to seek advice from doctors in 18 out of 45 comments, thus arthritis educators were behaving professionally and recognizing clinical scope of practice.

The content focus of discussion threads included symptoms, function, medication concerns, and the wider health care system. It is hypothesized that social media may be preferred for sharing minor concerns rather than serious symptoms or personal information [36]. This is likely because of the lack of anonymity of online social networks such as Facebook as all information is linked to a personal account. Nevertheless, sharing even minor concerns is likely to be of value to users of arthritis educator–led discussions as online social support groups have been identified as an important source of comradeship through sharing similar experiences [37]. Furthermore, participation in online communities for medical conditions can foster a sense of well-being and control and increase self-confidence and independence [37,38]. The content analysis identified multiple areas of health needs for the small number of people who did comment.

While the majority of interactions in arthritis educator–led discussions were between participants and arthritis educators, there was some peer-to-peer interaction. The online environment has potential for peer-to-peer support, including helping others understand medical science and care [39] and empowering others to find supportive and knowledgeable doctors [40,41]. Internet support groups have been suggested as able to mitigate the negative effects of time-pressured medical practice [39]. While peer-to-peer interaction may have benefits, within the context of this discussion facilitated by an arthritis educator of Arthritis NZ, the key drivers for the interactions are the program objectives of Arthritis NZ, in particular information and advice services.

### Limitations

The key limitations of this descriptive study include change in behavior as a result of being observed (Hawthorn effect) and the generalizability of the results. To collect data ethically, all users of the Arthritis NZ Facebook page were notified of the data collection before and during the study period, and posting comments implied consent. Arthritis educators and participants may have changed their posting behavior or/and users may have chosen not to post during the study period, introducing bias. Furthermore, the passive data collection and interpretation may not have accurately captured the participants’ intended meaning of their comment. In addition, when arthritis educator comments provided information, it is not possible to ascertain whether the information met the participants’ requirements.

The proportion of page followers who posted or commented during the 10 arthritis educator–led discussions in the study period was very low (5%) and most participants who did comment, did so infrequently. This study cannot infer anything about what benefit individuals may gain by reading arthritis educator–led discussions without posting.

### Conclusions

This study shows that a moderated discussion forum for people with arthritis can provide information and support to people affected by arthritis. An online information and advice service can be available to people who are unable or do not wish to attend face-to-face services or do attend formal health care services but have unmet or ongoing needs for information and support. Such an online forum could also be used to inform people of advances in treatments or available support services. The detailed and specific information that was requested by users does suggest that informational needs were not being met within formal health care settings and that behavior in online environments can provide insight into unmet health needs.

Users of online health forums could have the ability to connect with each other to exchange information and support, although this did not happen frequently in the current setting. Specific training for arthritis educators in posting behavior that engages users in discussion and facilitates peer-to-peer interaction could be encouraged. More active discussion may also occur in a less accessible discussion space, for example, a closed Facebook group, where the moderator of the group authorizes entry to the group and only users can view the comments. Furthermore, using people with arthritis as moderators may encourage active participation from more users or generate a richer discussion by bridging the space between laypersons and providers of health information. If these techniques encouraged more peer-to-peer interaction, a greater sense of community and comradeship for people affected by arthritis could be generated. Future research should focus on addressing barriers to user participation and assessing the impact of arthritis educator facilitation training, with the latter leveraging the Action Research paradigm.

## Appendix

Multimedia Appendix 1 Participant information available via Arthritis New Zealand Facebook page.

## Abbreviations

Arthritis NZ: Arthritis New Zealand
FDA: Food and Drug Administration
GPs: general practitioners
