# Supplementary material for: A Health Professional–Led Synchronous Discussion on Facebook: Descriptive Analysis of Users and Activities
Source: JMIR Form Res. 2017 Nov 22;1(1):e6. doi: 10.2196/formative.7257 (PMC6334677; doi:10.2196/formative.7257)
Supplement: Multimedia Appendix 1 [file formative_v1i1e6_app1.pdf]

Appendix 1 Participant information available via Arthritis New Zealand Facebook page

Pinned post: Arthritis New Zealand is interested in what you need from us so that we can improve our service to you. We are participating in research with Dr Rebecca Grainger from Otago University and Dr Karen Day from the University of Auckland. This involves analyzing the conversations that happen in our Facebook page, 1 October – 24 December 2015. Read more….*(link to PIS goes here)*

(*PIS link leads to text…*) Our research is titled ***'What can social media engagement tell us about unmet health needs for people affected by arthritis?'*** Thank you for showing an interest in this project. Please read this information carefully before deciding whether to participate or not. If you decide to participate we thank you. This information about our research has been written to help you decide if you want to participate.

Who is participating? Anyone who is following this Arthritis NZ page (if you clicked 'like' on the page you're a follower) and is participating in the online conversations that happen in the page are participants. The Arthritis Educators are also participating in the research. Arthritis NZ has granted permission for the research to be conducted.

How long will the research take? We aim to collect the discussion threads that happen between 1 October and 24 December 2015. Anything you contribute to the discussions during that time will be collected. During January and February a summer student will analyse the discussion content for comments that help us understand the needs of people affected by arthritis. The student will be supervised by Drs Grainger and Day.

Can I remain anonymous? Yes, you can. Someone from Arthritis NZ will collect the discussion threads from Facebook and will remove identifying information from the comments. The summer student and researchers will not view the Facebook page until after the analysis is complete.

What if I don't want to participate? If you don't want to participate at all, please don't comment in the Arthritis NZ Facebook page – you can remain in touch with the Arthritis Educator by phone and/or personal message via Facebook in the same way you would have done in Facebook. Alternatively, if you've decide that you want to withdraw a comment that you've made, you can remove it in the same way you usually delete comments in Facebook. Alternatively, you can indicate at the end of a comment 'not for research analysis' (abbreviated as "NFRA") and we will exclude it from our analysis. If you've been commenting during October – December and decide you want to withdraw all your comments please let the Arthritis Educator know (personal message via Facebook).

Will you tell us about the research results? Yes, we will write a summary report of our results and post it on the Arthritis NZ Facebook page for you to read and comment on. This will happen in March 2016. We will also write a report for Arthritis NZ to use to develop responses to any needs we identify in our research. We plan to write papers to publish in scholarly journals as well.

What do I do to participate in this research? Continue to post comments and questions and engage in discussions in the Arthritis NZ Facebook page as usual. You may initially feel a bit

uncomfortable about the scrutiny you know will happen when we collect the discussion threads for analysis. You may benefit from your contribution to the research as you reflect on your needs and post comments about them in Facebook, and gain a better understanding.

What will you do with the data? There are tools that analyse what kinds of people use a Facebook page. We will use one of these tools to identify patterns such as gender, age, and country, among the Arthritis NZ followers in Facebook. No identifying information will be available via this tool.

The content of the conversations will be thematically analysed in an Excel spreadsheet to identify needs. These needs will be compared with the Arthritis NZ services to identify gaps and derive responses. During the anonymising process, participants will be given an identification number so that we can match conversations and contributors to create a 'spider web' diagram of the nature, frequency, and directions of conversations to help analyse the value that these conversations add to needs identification.

How will the data be stored? The data collected will be securely stored in such a way that only those mentioned below will be able to gain access to it. Data obtained as a result of the research will be retained for **at least 10 years** in secure storage. No personal information will be held about participants.

How do I consent to participate? By continuing to participate in the Arthritis NZ Facebook page's discussions, you agree to participate in the research. By doing so you indicate that

- Your participation is voluntary (your choice)
- You understand that you can withdraw your contributions before 24 December when analysis will begin
- You can withdraw specific comments by deleting them or marking them for exclusion from analysis
- You are aware that you may initially feel uncomfortable about the potential scrutiny of your comments
- There is no compensation for your contribution to the research
- The results of the project may be published and will be available in the University of Otago Library (Dunedin, New Zealand) but every attempt will be made to preserve the anonymity of our Arthritis Educators and the followers of our Facebook page.

Who do I contact if I have any questions?

If you have any questions about our project, either now or in the future, please feel free to contact either:-

Dr Rebecca Grainger	Dr Karen Day
Department of Medicine, University of Otago Wellington	Department of Health Systems, The University of Auckland
University Telephone Number 04 385 5541	University Telephone Number: 09 737 7999
Email address: rebecca.grainger@otago.ac.nz	Email address k.day@auckland.ac.nz

This study has been approved by the Department stated above. However, if you have any concerns about the ethical conduct of the research you may contact the University of Otago Human Ethics Committee through the Human Ethics Committee Administrator (ph 03 479-8256). Any issues you raise will be treated in confidence and investigated and you will be informed of the outcome.
